# Benefit of Shading by Nurse Plant Does Not Change along a Stress Gradient in a Coastal Dune

**DOI:** 10.1371/journal.pone.0105082

**Published:** 2014-08-15

**Authors:** Camila de Toledo Castanho, Paulo Inácio Prado

**Affiliations:** Departamento de Ecologia, Instituto de Biociências, Universidade de São Paulo, São Paulo, Brazil; Beijing Forestry University, China

## Abstract

The proximity of adult neighbors often increases the performance of woody seedlings under harsh environmental conditions but this nurse plant effect becomes less intense when abiotic stress is alleviated, as predicted by the stress gradient hypothesis (SGH). Although some studies have tested how the net nurse effect is changed by stress, few studies have tested how the mechanism that drives the facilitative effect of nurse responds to changes in stress. We conducted field experiments in a subtropical coastal dune to test if shading drives the known nurse effect of adults of the tree *Guapira opposita* on seedling performance of another tree species, *Ternstroemia brasiliensis*. We transplanted *T. brasiliensis* seedlings to three neighbor environments: under a *G. opposita* crown, under artificial shade and without neighbor as a control. Furthermore, assuming that proximity to the seashore correlates with stress intensity, we tested if the potential shade-driven facilitation became less intense as stress decreased. Regardless of the proximity to the seashore, after a year, the survival of *T. brasiliensis* seedlings was twice as high when the seedlings were under *G. opposita* or under artificial shade compared to the control, indicating that the nurse effect is driven by shade and that this facilitation mechanism is constant along the stress gradient. However, *G. opposita* and artificial shade had a negative effect on seedlings growth. Overall, our results showed that the facilitation mechanism behind the nurse effect did not wane as the stress was reduced. Furthermore, in spite of the potential costs in terms of biomass production, our study highlights the potential of nurse plants and artificial shade as techniques to improve the survival of transplanted seedlings used in the restoration of degraded shrubland coastal dunes.

## Introduction

Facilitation occurs when the fitness of an individual increases due to the presence of another individual of the same trophic level [1]. Facilitation between plants has been well demonstrated in a variety of systems, from deserts to forests [2], [3]. The emblematic type of facilitative interaction in plant communities is the nurse plant effect, in which adults improve the performance of young individuals of the same species or other species that grow in their vicinity [4], [5]. Some of the potential mechanisms behind the nurse plant effect are amelioration of the microclimatic conditions, increases in nutrients and soil moisture and protection from herbivores [6]–[9]. The establishment of stress-sensitive species facilitated by nurse plants has the potential to affect species diversity [10], [11] and in some cases the succession of plant communities [8], [12], [13]. The nurse plant interaction also has practical applications; for example, it may be used as a tool to improve the survival of transplanted seedlings in the restoration of degraded areas [5], [14].

Theoretical models predict that the net interactions between plants are affected by environmental conditions and that the importance and intensity of facilitation decreases as the conditions become less severe, a proposition known as the stress gradient hypothesis (SGH) [15], [16]. Indeed, most observed cases of nurse plant interactions have occurred in harsh environments, such as arid and semi-arid biomes [4], where the alleviation of environmental severity, such as the increase in water availability, tends to reduce the net positive effect of the nurse plant [5], [17]. Because the net interaction includes positive and negative interactions, the reduction in the net positive effect as the stress decreases can be reached through an increase in the competitive interactions and/or a reduction in the positive effects of the nurse plants. Although several studies have tested how the net nurse effect changes with stress level, few studies have assessed how specific facilitative mechanisms change with the environmental conditions (but see [18], [19]). This knowledge is essential to deepen our mechanistic understanding of the effects of abiotic stress on the net interactions between plants.

Coastal dunes are harsh environments in which stress and disturbance by poor soil nutrients, salt spray and wind decrease from the seashore towards the inland areas, producing a clear environmental gradient [20], [21]. These features make this environment a suitable system to test predictions regarding the stress gradient hypothesis. Several studies conducted on coastal dunes have shown that initial colonizers can act as nurse plants of woody species [7], [13], [22], [23], indicating that facilitation plays an important role during the primary succession and transition from a herbaceous to a woody vegetation state. Some studies have demonstrated that microclimatic conditions are less severe under the shade of nurse plants than in the gaps between these plants, suggesting that the shade provided by the nurse plant is a proximate driver of facilitation in dunes [22]–[24]. Indeed, as has been experimentally demonstrated in a variety of systems, the shade provided by the nurse plant canopy may facilitate woody seedlings by more than one mechanism, such as the direct amelioration of microclimatic conditions and/or an increase in the soil water availability [9], [18], [19], [25]. Facilitation may also occur indirectly due to the nurse plants limiting the biomass of highly competitive herbs [26]. However, the studies conducted in dunes consist of correlative observations that the environmental conditions are better under the shade of nurse plants than in the gaps between them [22]–[24]. These studies generated a viable hypothesis but did not prove that the shade of the nurse plant drives positive plant interactions in harsh environments (but see Shumway [7] for shade drive facilitation on herbaceous species). The lack of conclusive evidence that the facilitation between adult plants and woody seedlings in dunes is driven by shade is rather surprising, given the potential benefits of shade in an environment where the high soil temperatures coupled with low moisture create rather inhospitable conditions for seedling survival [27].

In Brazilian coastal dunes (locally called *restinga* vegetation), facilitation has been proposed as a key ecological process [28], and recent studies demonstrated nurse plant effects in this vegetation [29], [30]. Lower temperatures and less intense photosynthetic active radiation (PAR) occur beneath nurse plants compared to sites without vegetation, indicating that the shade provided by the nurse canopy is potentially responsible for the nurse effect (unpublished data, C.T. Castanho et al.). In this study, we performed transplant experiments along a natural stress gradient in coastal dunes from southeast Brazil to test the following hypotheses: (1) shading is a facilitative mechanism that allows mature trees to provide a nurse effect, and (2) the net outcome of the nurse interaction and the shade-driven facilitation change along a natural gradient of environmental severity. We transplanted seedlings of a facilitated tree species under a nurse plant, under artificial shade, and in open microsites as a control, and we compared seedling survival and growth after one year. We expected: (1) higher seedling performance (survival and growth) under the nurse plant and artificial shade than in the open microsites and (2) that the net interaction and the positive effect of shade would increase with proximity to the seashore.

## Methods

### Ethics Statement

The research permission to conduct the field study in the Ilha do Cardoso State Park, a protected area, was approved by IF/SMA - Instituto Florestal/Secretaria do Meio Ambiente (permission SMA 000.541/2008).

### Study Site

We conducted this study along a beach-inland gradient located on the southern shore of Ilha do Cardoso State Park, a coastal island reserve in southeastern Brazil (25°12'S, 44°17'W). The mean annual precipitation in Ilha do Cardoso is 2050 mm, with almost 70% of the rain falling between November and April (Figure S1). The mean annual temperature ranges from 18 to 26°C, with mean monthly maximum reaching 30°C in February and mean monthly minimum reaching 13°C in July.

Sand dune vegetation in southeastern Brazil is formed by a mosaic of plant communities that occupy sandy plains formed by marine deposits in the late Quaternary and range from pioneer herbaceous vegetation to forests [31]. The dune system in the study area is formed by parallel ridges that occupy an approximately 400 m strip of land between the ocean and an inland estuary. Along this beach-inland gradient, there are several parallel ridges where the vegetation ranges from creeping psammophytes to open scrub vegetation to a strip of forest on the inland-most part. Between the ridges are the dune slacks, which are depressions flooded by fresh groundwater during the summer. Along the beach-inland gradient, vegetation cover and biomass on the ridges increase with distance from the seashore. This vegetation pattern, a common feature in coastal communities [32], reflects the decrease in stress and disturbance intensity as the proximity from the shore decreases [33] and also the time available for succession when shoreline accreation is occurring [34]. We conducted the experiments in an area with open scrub vegetation (the intermediate part of the gradient) that is composed of several ridges with 1–3 m sparse trees, shrubs and woody clumps of various sizes, and a perennial herbaceous cover on the sandy substrate. Our study was conducted in this part of the gradient because isolated adult trees occur in this region, allowing us to test if shading by individual trees provides a nurse effect.

### Study Species

To test our hypotheses, we used two treelet species common in coastal dunes from southeastern Brazil: *Guapira opposita* (Vell.) Reitz (Nyctaginaceae) as the benefactor and *Ternstroemia brasiliensis* Cambess. (Pentaphyllacaceae) as the beneficiary. We chose this pair of species because a previous study conducted at the same study site demonstrated a nurse plant effect of *G. opposita* on the survival of *T. brasiliensis* seedlings (unpublished data, C.T. Castanho et al.). *Guapira opposita* is a tree, or treelet, that is widely distributed across Brazilian ecosystems [35]. In the study area, it is found in regions ranging from open scrub to forest. In the open scrub vegetation, where the experiments were carried out, *G. opposita* adults are relatively abundant and shorter (on average 1–2 m in height) than those found in forests, most likely due to harsh abiotic conditions. *Ternstroemia brasiliensis* is a Brazilian endemic tree relatively common on coastal plains and in high-altitude forests [36]. *Ternstroemia brasiliensis* is a common species in the study area and also occurs in regions ranging from open scrub to forests.

### Experimental Design

**Figure 1:**
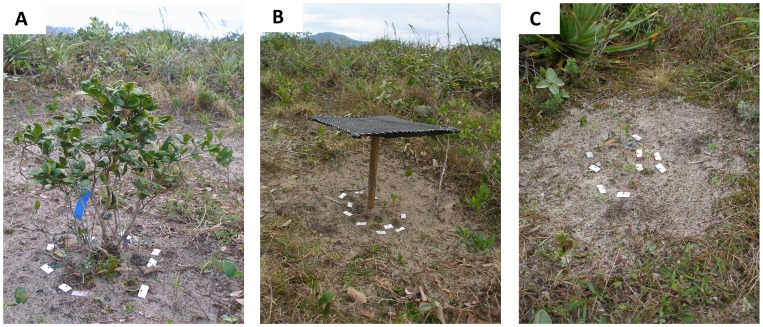
Three levels of the neighbor factor implemented to investigate if shading is the mechanism that causes adults of *Guapira opposita* to provide a nurse effect on survival of *Ternstroemia brasiliensis* seedlings in coastal dune vegetation in São Paulo, Brazil. A) beneath a *G. opposita* canopy; B) beneath artificial shade provided by a mimic of *G. opposita* shade; and C) control with no neighbor and no shade. In the three levels of neighbor, it is possible to see the seedlings of *Ternstroemia brasiliensis* with their metallic tags. All other plants in a 0.5 m radius were regularly clipped.

To test our hypotheses we set 45 experimental blocks in the scrub vegetation to evaluate the performance of transplanted *T. brasiliensis* seedlings. To test if shading is the mechanism that allows *G. opposita* to provide a nurse effect, each block consisted of three levels of a neighbor factor: underneath *G. opposita* adult, underneath artificial shade provided to mimic *G. opposita* shade, and without any shade or plant as a control (Figure 1). To set up the experimental blocks, we haphazardly selected 45 *G. opposita* adults with similar heights (0.78±0.16 m; mean ± sd) and canopy areas (0.48±0.21 m^2^; assuming an ellipse) along the open scrub vegetation. The artificial shade and control areas were 2 m from the canopy of the *G. opposita* trees, each in a random direction centered in the *G. opposita* tree. If the selected site was occupied by an adult tree or exhibited marked soil differences, we randomly chose another microsite. We regularly clipped all other plants in a 0.5 m radius at the ground level in the three levels of the neighbor factor (Figure 1).

The artificial shade was designed to mimic *G. opposita* shade and consisted of a rectangular bamboo frame covered with black nylon mesh (70% shadow) that was suspended above the ground with a bamboo stick. Considering that the objective of the artificial shade was to simulate the shade of the paired *G. opposita* tree, both the frame area and stick height of each artificial shade structure were equal to the area and height of the *G. opposita* nurse tree of the same experimental block. To test how stress affects the potential shade facilitative effect of *G. opposita* on *T. brasiliensis*, we assumed that the proximity to the seashore correlated with stress intensity and placed the 45 experimental blocks at three different proximity classes to the shore (proximity I = 40–50 m from the seashore; proximity II = 120–130 m; proximity III = 190–200 m; Figure S2).

In March 2009, we collected *T. brasiliensis* seedlings that were naturally occurring at the study site and transferred them into 43 cm^3^ plastic pots (28 mm in diameter and 115 mm in height) with a mixture of 1∶1 dune soil:organic compost Biomix. We kept the seedlings in a greenhouse located in the northeastern section of Ilha do Cardoso State Park until the beginning of the experiment. On 24–25 Jun 2010, we transplanted 1350 seedlings 6.6±1.6 cm (mean ± sd) in height to the experiment location. We planted 30 randomly selected *T. brasiliensis* seedlings in each experimental block (10 per neighbor level; Figure 1). In the following days, we watered the seedlings to avoid transplant shock. Assuming that seedling death on the days following transplantation was the result of transplant shock, we replaced 95 dead seedlings on 12 Jul 2010, watered all the seedlings again and started the experiment. In summary, our experimental design included 15 experimental blocks per seashore proximity and each block included the three levels of neighbor factor. There were 10 seedlings per neighbor level, for a total of 1350 *T. brasiliensis* seedlings (15 blocks × three proximities to the shore × three neighbor levels ×10 seedlings).

At the start of the experiment, we measured the height, the diameter at ground level and the leaf number of each seedling. To calculate the increase in biomass over the course of the experiment, we estimated the initial dry biomass of the experimental seedlings using an allometric equation based on a subsample of 60 seedlings for which height, diameter at ground level, leaf number and dry biomass were measured. We decided to use only the aboveground biomass to make inferences regarding *T. brasiliensis* growth because despite being careful in the removal of each seedling from the field at the end of the experiment, some fine root material was likely lost. Adopting a stepwise Akaike information criterion (AIC) procedure, as explained in detail in the next subsection, we selected the best allometric model for calculating the initial aboveground biomass of *T. brasiliensis* seedlings. The selected equation included height (h) and diameter at ground level (d) and had an R^2^ = 0.42: E[IAB] of *T. brasiliensis* = −0.18+0.09d+0.03h, where E[IAB] is the expected initial aboveground biomass (g).

We monitored seedling survival monthly until July 2011 for a total of 358 days. At the end of the experiment, we harvested all the surviving seedlings to calculate the final aboveground biomass. We gently washed the harvested seedlings and dried them at 62°C to reach a constant mass.

To understand how shade changes the environmental conditions, we measured the temperature and photosynthetically active radiation (PAR) under the three levels of neighbor factor in 18 of the 45 experimental blocks (three levels of neighbor ×18 replicates = 54 observations). We took surface soil temperatures using a UT301C infrared thermometer gun (UNI-TREND, Kowloon, Hong Kong) and measured PAR using a 3415FSE solar quantum meter (Spectrum Technologies, Plainfield, USA). Each observation was the average of three measurements to compensate for heterogeneity, especially under the *G. opposita* canopy. We collected the measurements between 12:00 and 01:00 PM during a sunny day in December 2011.

### Statistical analysis

To test if the survival of *T. brasiliensis* seedlings was higher under *G. opposita* and artificial shade than in open sites and if this effect shifted along the environmental gradient, we used mixed effects models to consider the random effects of the blocks. We used generalized linear mixed effect models (GLMM) to model the proportions of surviving seedlings after one year as linear responses, assuming a binomial error distribution with the logit link function. The expected logit of survival proportion was modeled as a function of two fixed effects and their interaction (presence of a neighbor and proximity to the seashore) and one random effect (the block). To test the effects of the neighbor and proximity to the seashore on *T. brasiliensis* seedling growth, we used linear mixed effect models (LMM). The aboveground biomass increment was analyzed as a function of the fixed variables neighbor and proximity to the seashore and their interaction, and the random variables block and neighbor nested in block. For the growth data, we also modeled the variance structure to account for heteroscedasticity among the data collected at distinct proximities from the shore (Appendix S1). Finally, to test the effect of the neighbor on abiotic parameters, we used LMM. In this case, the soil temperature and PAR were modeled as a function of the fixed variable neighbor and the random variable block.

To evaluate the importance of each fixed effect in each model described above, we selected the optimal fixed structure, as well as the random structure of each model, following the top-down strategy recommended by Zuur et al. [37]. Although we are interested in the fixed factors, the selection of the appropriate random structure is very important because it affects the estimates of the fixed effects [37]. Therefore, we first selected the appropriate random component, that is, whether the random variable influences the neighbor effect (model slope) or only the intercept. Once the optimal random structure was found, we proceeded with the fixed structure selection. We compared models with and without each fixed factor. The full model included the single terms (the neighbor and the proximity to the shore for survival and growth and only the neighbor for abiotic parameters) and the interaction between neighbor and proximity (for survival and growth), as well as the random component. The comparison was done hierarchically; thus, progressively simpler models were fitted by dropping the interaction first, then each single term. In all models, including the model without any fixed term, the random effect was retained. Diagnostics and validation of the selected models are presented in Appendix S1.

For statistical inferences, we employed model selection using Akaike's information criterion (AIC), which is a likelihood-based measure of model plausibility that penalizes more complex models, i.e., those with a greater number of parameters [38]. We ranked the models using AIC such that the best model, the one that sacrifices the least information when it is used to approximate the truth, had the lowest AIC value [39]. The differences between the AIC value of the best model and the values of each model ranked below it (ΔAIC = AIC of a given model – AIC of the best model) provide information for evaluating which models in a set are the best. Values of ΔAIC between 0 and 2 indicate similar support for the two models [38]. We used the R environment (version 2.14.1, R Development Core Team 2011; R Foundation for Statistical Computing, Vienna, AT) with the packages lme4 and nmle for all statistical analyses.

## Results

### Survival

Of the 1350 *T. brasiliensis* seedlings monitored, 404 (30%) survived after 358 days (Figure S3). The model selection indicates that the random structure of the best model included both the intercept and slope components (Table S1), which means that in addition to the great variability in seedling survival among the blocks, the neighbor effect also varied among the blocks (Figures S4 and S5).

**Figure 2:**
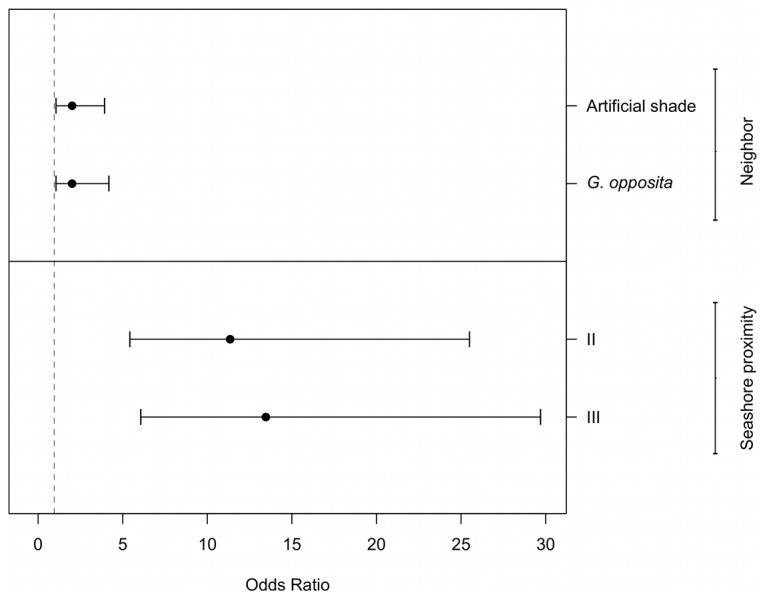
Odds ratios (95% confidence intervals) of seedling survival of *Ternstroemia brasiliensis* after 358 days. The odds of seedling survival beneath *Guapira opposita*, artificial shade and seashore proximity II and III was compared to seedling survival in the reference condition (control with no neighbor in the seashore proximity I, the closest proximity to the seashore). An odds ratio of 1 indicated no difference between odds of seedling survival in the treatment and in the reference; >1 indicated a greater survival odds in the treatment than in the reference. The estimates are based in the selected model (model M2.S in Table 1).

Despite the variation among treatments due to random effects, the selected model indicates that neighbor presence, as well as the proximity to the shore, affected the survival of *T. brasiliensis* seedlings (Table 1; Figure 2). The odds of seedling survival beneath a *G. opposita* crown or artificial shade were twice that of seedlings in open microsites (the odds ratio was calculated as the exponential of the fixed term, *e* ^0.73^ = 2.08). No consistent difference was identified between seedlings under *G. opposita* and the artificial shade (Figure 2). As expected, the proximity to the shore negatively affected seedling survival. The odds of seedling survival were, on average, 11 times higher at proximity class II (*e* ^2.43^) and 13 times higher at proximity class III (*e* ^2.59^) in comparison with class I (Figure 2). The absence of the interaction term between neighbor and proximity in the selected model indicates that the effect of neighbor presence did not change along the environmental gradient, which does not support the predictions of the stress gradient hypothesis.

**Table 1:** Model selection results for the survival and growth of *Ternstroemia brasiliensis* seedlings.

	Predicted terms included				
Models	Fixed	Random	K	AIC	Δ AIC
SEEDLING SURVIVAL					
M1.S	NE+SEA+NE:SEA	1+NE | Block	15	301.5	3.0
**M2.S**	**NE+SEA**	**1+NE | Block**	**11**	**298.5**	**0.0**
M3.S	NE	1+NE | Block	9	301.1	2.6
M4.S	SEA	1+NE | Block	9	339.3	40.7
M5.S		1+NE | Block	7	342.3	43.8
SEEDLING GROWTH					
M1.G	NE+SEA+NE:SEA	1|Block/NE	14	124.1	5.3
**M2.G**	**NE+SEA**	**1|Block/NE**	**10**	**119.8**	**1.0**
**M3.G**	**NE**	**1|Block/NE**	**8**	**118.8**	**0.0**
M4.G	SEA	1|Block/NE	8	131.7	12.9
M5.G		1|Block/NE	6	130.3	11.5

For survival, the models are general linear mixed models (GLMM) of the proportion of surviving seedlings after one year as a binomial response.

For growth, the models are linear mixed models (LMM) and increase in aboveground biomass after a year as a Gaussian response. Neighbor presence (NE), proximity to the seashore (SEA), and their interaction (NE:SEA) are the fixed variables, and block is the random variable. For each response variable all models include the same random effects structure. The selected models (Δ AIC<2) are in bold.

### Growth

**Figure 3:**
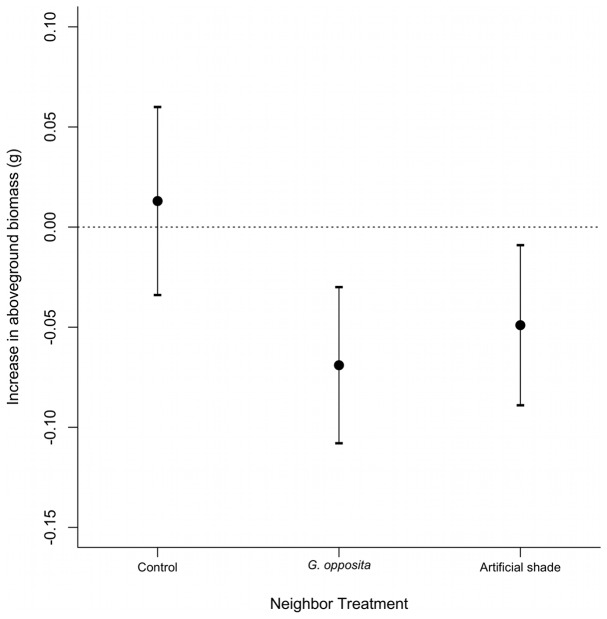
Growth of *Ternstroemia brasiliensis* seedlings after 358 days in the three levels of the neighbor treatment (mean±95% confidence interval). The estimates are based in the selected model (model M3.G in Table 1).

There was a negative effect of *G. opposita* and artificial shade on the growth of *T. brasiliensis* seedlings, regardless of the proximity to the seashore (Table 1). While in the exposed microsites no growth was observed after a year, beneath natural and artificial shade the seedlings decreased their aboveground biomass, on average to 34% and 24%, respectively, of their initial biomass (Figure 3). There is no conclusive evidence that proximity to the seashore influences the growth of *T. brasiliensis* seedlings. The model that included proximity and neighbor presence was as plausible as the model that included only neighbor presence (Table 1 and Table S1).

### Abiotic parameters

Both *G. opposita* and artificial shade strongly buffered soil temperature and PAR at midday (Table S2 and Table S3). Compared to the open microsites, the soil temperatures were, on average, 34% and 42% lower under *G. opposita* and artificial shade, respectively (Table 2). The reduction in PAR reached 85% under *G. opposita* and 75% under artificial shade (Table 2).

**Table 2:** Soil temperature and PAR (mean±1 SE) during the noon period on December 23, 2012 in the control (open microsites), under a *Guapira opposita* crown and under artificial shade (n = 18).

	Control	G. opposita	Artificial shade
Temperature (°C)	63.7±0.73	42.1±0.93	36.8±0.93
PAR (µmol m^−2^ s^−1^)	2000±13	297±18	492±18

## Discussion

We found that *G. opposita* trees have a positive net effect on the survival of *T. brasiliensis* seedlings, which was equivalent to the effect of artificial shade. Therefore, the shade provided by the *G. opposita* crown is a proximate cause of the positive net effect of *G. opposita* on the survival of *T. brasiliensis* seedlings. Furthermore, the benefits provided by *G. opposita* and the artificial shade were constant along the environmental gradient, contrary to our expectations.

Shade-driven facilitation by woody species on tree seedling survival has been experimentally detected in other harsh natural systems, such as temperate open woodlands [9], [18], [40] and lake sand dunes [41], as well as in degraded systems undergoing restoration [42]. Shade provided by woody species protects seedlings from lethal temperatures, reduces photoinhibition and in some cases improves soil water through reductions in transpiration losses and soil moisture evaporation [3], [7], [42]. Most plants suffer physiological damage at temperatures higher than 50°C as a consequence of the degradation of enzymes and cell membranes [43]. We found that during the hottest period of a summer day, the soil temperatures reached more than 60°C in open microsites, whereas the shade provided by *G. opposita* and the artificial source efficiently buffered extreme temperatures to below the critical temperature and reduced the overabundant amount of light typical of midday. Assuming that the artificial shade effectively simulated the shade provided by the *G. opposita* trees, our results conclusively showed that shade is a proximate driver of the nurse effect of *G. opposita*. A promising direction for this research is to develop an understanding of the ultimate mechanisms behind this shade-driven facilitation.

Although plant-plant interactions are frequently species-specific [44], the shade-driven nurse plant effect found between *G. opposita* and *T. brasiliensis* may be a common phenomenon among woody adults and seedlings of other species in coastal shrublands. In these open ecosystems, woody plant adults usually provide shade regardless of the species identity. At the same time, as the early phase of life is the most vulnerable phase to harsh abiotic conditions, woody plant seedlings may benefit from the environmental amelioration provide by shade, regardless of the seedling species. Accordingly, the positive effects of the shade provided by adults to woody seedlings is common in a variety of shrubland ecosystems under harsh conditions [9], [18], [25], [42], [45]. Similar experiments with other species are an obvious way to confirm whether nurse effects due to shade are widespread among woody species in coastal dunes. Nevertheless, a more effective and mechanistic approach is to investigate which traits of the nurse and beneficiary make this positive interaction mechanism more likely [46].

**Figure 4:**
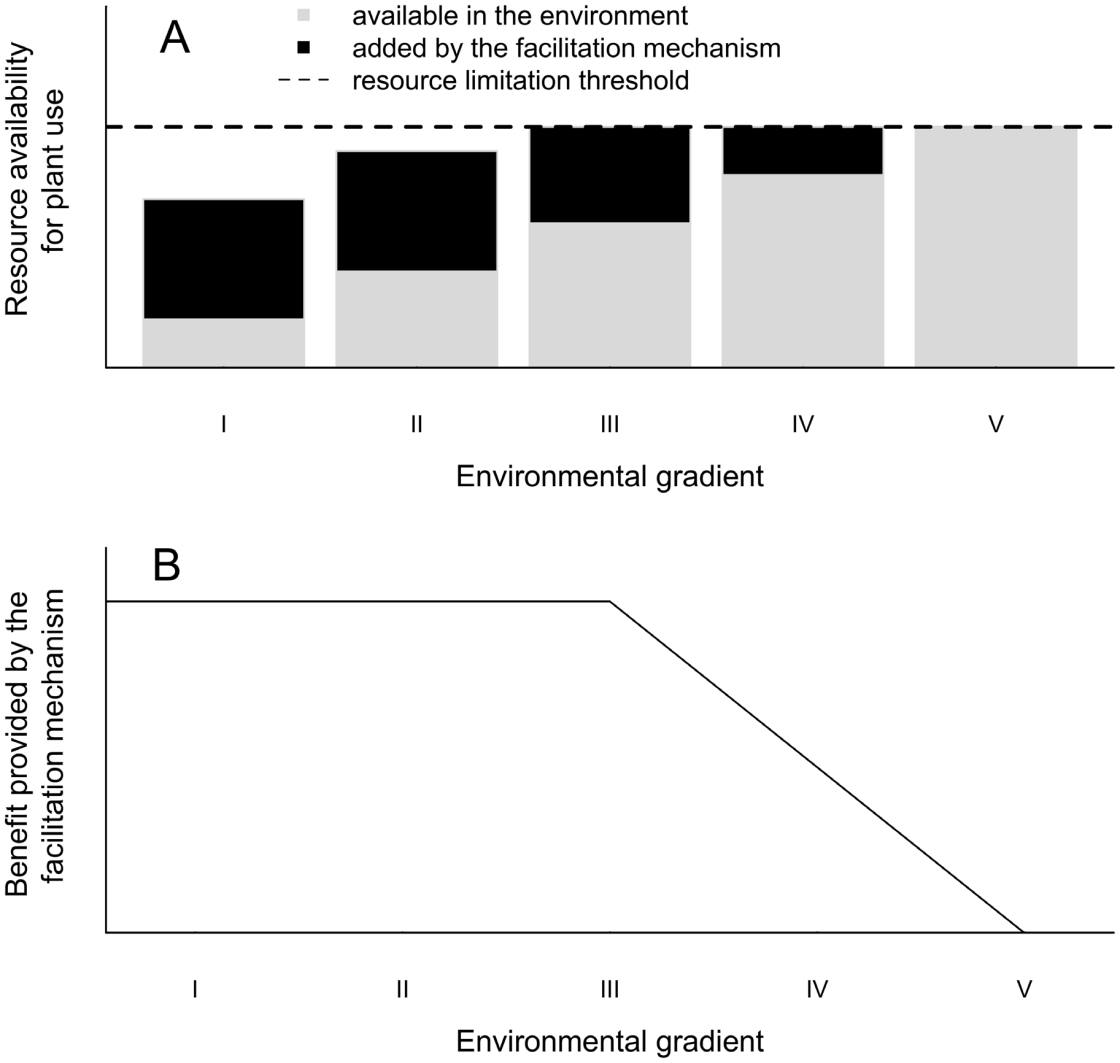
A graphical model of the stress gradient hypothesis regarding facilitation mechanism, which shows how a constant facilitation effect can occur along an environmental gradient. A) A gradient is defined by the monotonic variation in the availability of a resource (gray bars), which increases by a constant amount due to a plant facilitation mechanism (black bars). The dashed line represents the resource limitation threshold: below this line, the resource is limiting, and above this line, the resource is not limiting. B) The relationship between the environmental gradient (as described for graph A) and the benefits provided by the additive facilitation effect. Note that in the region of the gradient where the resource is limiting (levels I and II in graph A), the benefits provided by the facilitator are constant along the environmental gradient (levels I and II in the graph B). However, from the point where the total availability of the resource (from the environment and the facilitator) reaches the resource limitation threshold (gradient level III), further increases in the resource availability along the gradient will reduce the portion of the resource used by the plant that is provided by the facilitator. As a consequence, in this region of the gradient (levels III–V), the benefits provided by the facilitator wane as the stress is reduced.

Environmental conditions are critical factors that influence the net interactions between plants. Theoretical models state that the importance of facilitation and competition will vary inversely across stress gradients, with facilitation being dominant in harsh conditions [15], [16]. We found a marked decrease in the survival of *T. brasiliensis* towards the shore, confirming our assumption that proximity to the shoreline is a good surrogate for stress in sand dunes. Indeed, the use of the organism performance to estimate stress is suggested, as it is more appropriate than the direct measurement of environmental conditions, especially when multiple stress factors occur simultaneously [47]. However, contrary to our expectations, we did not find any influence of proximity to the shore on the nurse effect of *G. opposita* and the artificial shade on seedling survival, indicating that the shade-driven facilitation of *G. opposita* remained constant upon variation in environmental conditions. The explanation of this apparent contradiction could be that the stress factor ameliorated by the nurse plant shade remains limiting along the entire gradient. Direct facilitation occurs when the neighboring plant ameliorates a condition or resource that limits plant performance [3]. Therefore, a reduction in the facilitation intensity should occur when the stress factor alleviated by the facilitator decreases along a gradient, making the facilitation benefit irrelevant. However, if the stress factor alleviated by the facilitator remains limiting even with the reduction in environmental severity (Figure 4A), the benefit provided by the facilitator will be constant (Figure 4B). Therefore, our explanation for the shade-driven facilitation being independent of stress is that the stress factor alleviated by the nurse shade remains limiting along the environmental gradient, even with the benefit provided by the nurse. Assuming, for example, that the shade-driven nurse effect improves water availability, if our hypothesis is correct, we expect that water addition would not change the benefits provided by the shade while water remains a limiting resource. When the water added in the soil summed with the water retained because of the nurse's presence reaches the content required by the beneficiary, then we expect a reduction in the intensity of the shade-driven nurse effect. One of the challenges in testing this hypothesis is to consider a fine-scale variation in the stress factor ameliorated by the facilitator in a range wide enough to encompass limiting and non-limiting availability of the stress factor.

Despite the shade-driven facilitation of seedling survival, *G. opposita* and the artificial shade had negative effects on seedling growth. The distinct effect of shade on the performance of the seedlings indicates that the conditions and resources that were ameliorated by the shade and positively influence survival do not necessarily affect growth [48], [49]. In addition to the potential positive effects on microclimate amelioration and water availability, shade can also have negative effects, including limiting light availability for photosynthesis [3]. However, light limitation does not fully explain our results because even without light limitation (exposed sites), no biomass increase was observed after a year. In addition, light limitation under the shade provided by both *G. opposita* and the mimic seems implausible because the shade was effective only around midday when the leaves were exposed to a maximum amount of light. Even under shade, light availability at this time is much higher than the compensation point for sun plants, which ranges from 10 to 20 µmol m^−2^ s^−1^ [50]. *Ternstroemia brasiliensis* seedling growth could have been so slow that we did not detect biomass increases in the one-year experiment. In this case, how do we explain the reduction in aboveground biomass in the seedlings under shade? The reduction in biomass after a year could be the result of the slow growth of the species combined with higher herbivory under shade. In wooded pastures, the removal of seeds is higher under shrubs than in open areas, primarily due to insects [51]. Herbivore arthropods may prefer to forage under shade because of the milder microclimatic temperature, a hypothesis to be tested in future studies.

Coastal sand dunes are harsh environments in which plant establishment and growth are limited by soil moisture, soil nutrients, wind exposure, salt spray, sandblasting, soil salinity and burial [20], [52], [53]. As observed in arid systems [54], several studies have highlighted the role of adult woody species in nucleating the settlement of woody seedlings in coastal dunes [22], [28], [30], [41], [55]. The main mechanisms behind this nucleation process are the attraction of dispersers [22] and facilitation mechanisms such as environmental amelioration provided by shade, as demonstrated in the present study (see also [7], [41], nutrient improvement [7], [22], [56] and reductions in wind speed and sand movement [23]. The positive feedback created by the first woody individuals reinforces the woody state, which is a key process for the successional transition from herbaceous to woody physiognomies [22], [28], [30], [55]. This positive feedback between woody individuals could be of great value in successfully restoring shrublands and forests. Accordingly, increasing the populations of nurse plants has been considered a promising strategy for restoring degraded woodlands [5], [49]. However, the dependence on nurse-plants to make environmental conditions suitable for woody plant establishment implies that the lack of at least some adult woody individuals hinders sustained revegetation [54], making the degraded system resilient to restorative change [57]. Remaining treelets (such as the isolated *G. opposita* individuals used in our experiment) in degraded areas have high restoration value because they can nurse transplanted seedlings used in restoration programs, thus triggering the transition from an herbaceous state to a woody state. Despite the potential negative effect on biomass production, the use of nurse plants is considered a promising technique to increase survival in restoration programs because seedling survival in recovering degraded systems is quite low [5], [42], [49], [58]. Therefore, traditional techniques that remove all pre-existing woody vegetation before planting a desired species may be suboptimal when the objective is to restore a shrubland or a forest [49]. Our study also suggests that in the absence of remaining vegetation, artificial shade structures that simulate the beneficial effects of nurse plants could be an effective alternative for increasing the establishment of transplanted seedlings in degraded shrubland coastal dunes.

In conclusion, our study indicates that the facilitative interactions in a shrubland coastal dune are driven by the shade provided by the nurse plant. Contrary to the predictions of the stress gradient hypothesis, neither the net nurse plant interaction nor the shade-driven facilitation changed along the beach-to-inland stress gradient. This result suggests that the stress level itself is not a good predictor of the facilitation mechanism intensity, most likely because the stress factor alleviated by the interaction remains limiting for the beneficiary along the environment gradient. Our results highlight the importance of the management of nurse plants and the use of artificial shade structures to increase the establishment of woody seedlings in restoration programs of shrubland coastal dunes.

## Supporting Information

Figure S1 **Monthly precipitation in the region of the study site.**
(DOC)

Figure S2 **Open scrub vegetation located at Ilha do Cardoso State Park, São Paulo, Brazil.**
(DOC)

Figure S3 **Daily mortality rate of** ***Ternstroemia brasiliensis*** **seedlings per time interval along all the experiment.**
(DOC)

Figure S4 **Proportion of surviving seedlings of** ***Ternstroemia brasiliensis*** **at three levels of the factor proximity to the seashore (I - closest; III – farthest) and three levels of the factor neighbor.**
(DOC)

Figure S5 **Random effects and their 95% interval on survival of** ***Ternstroemia brasiliensis*** **seedlings in the three levels of the neighbor treatment (control, under** ***Guapira opposita*** **and under artificial shade) in each experimental block.**
(DOC)

Table S1 **Results of random component model selection for survival and growth of** ***Ternstroemia brasiliensis*** **seedlings.**
(DOC)

Table S2 **Results of the model selection for soil temperature and photosynthetically active radiation (PAR).**
(DOC)

Table S3 **Results of random component model selection for soil temperature.**
(DOC)

Appendix S1 **Statistical models - full specification, diagnostics and validation.**
(DOC)

Dataset S1 **Abiotic parameters and the survival and growth of the** ***Ternstroemia brasiliensis*** **seedlings 358 days after transplantation.**
(XLS)
