# Supplementary material for: Benefit of Shading by Nurse Plant Does Not Change along a Stress Gradient in a Coastal Dune
Source: PLoS One. 2014 Aug 15;9(8):e105082. doi: 10.1371/journal.pone.0105082 (PMC4134255; doi:10.1371/journal.pone.0105082)
Supplement: Figure S1 — Monthly precipitation in the region of the study site. (DOC) [file pone.0105082.s001.doc]

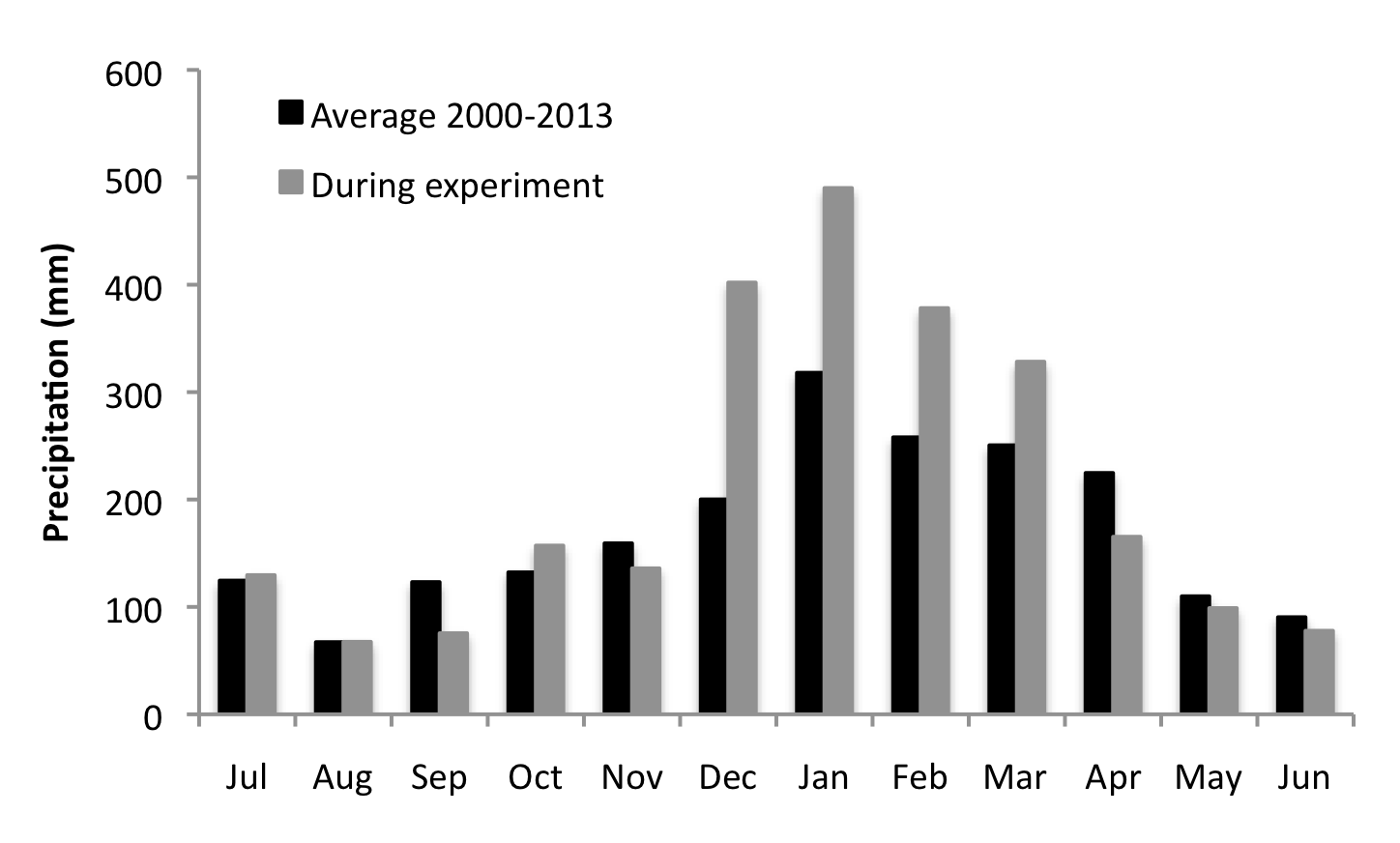


**Figure S1 Monthly precipitation in Cananéia, town where Ilha do Cardoso State Park is located.** The black bars show the average monthly precipitation based on the last 13 years (from 2000 to 2013) and the gray bars show the monthly precipitation during the experiment that lasted from 13 July 2010 to 06 July 2011 (Source: http://www.ciiagro.sp.gov.br/ciiagroonline/Quadros/QChuvaPeriodo.asp).
