# Supplementary material for: Benefit of Shading by Nurse Plant Does Not Change along a Stress Gradient in a Coastal Dune
Source: PLoS One. 2014 Aug 15;9(8):e105082. doi: 10.1371/journal.pone.0105082 (PMC4134255; doi:10.1371/journal.pone.0105082)
Supplement: Figure S2 — Open scrub vegetation located at Ilha do Cardoso State Park, São Paulo, Brazil. (DOC) [file pone.0105082.s002.doc]

**
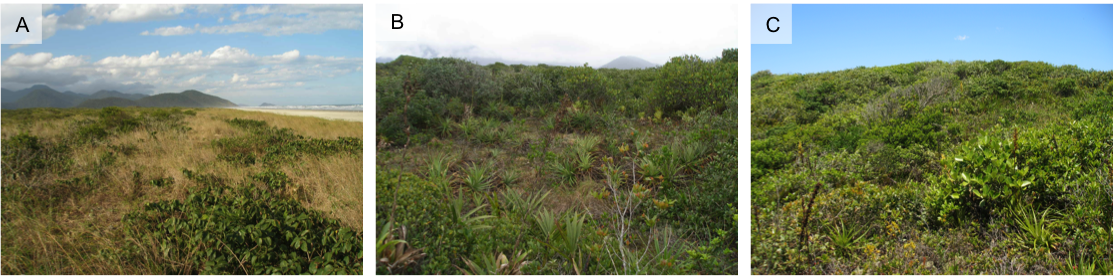
**

**Figure S2** **Open scrub vegetation located at Ilha do Cardoso State Park, São Paulo, Brazil.** The three images illustrate the vegetation in three points along the beach-to-inland gradient: A) Vegetation located around 40-50 m to the seashore (proximity I to the seashore in our models); B) Vegetation located between 120-130 m to the seashore (proximity II) and C) Vegetation located between 190-200 m to the seashore (proximity III).
