# Supplementary material for: Benefit of Shading by Nurse Plant Does Not Change along a Stress Gradient in a Coastal Dune
Source: PLoS One. 2014 Aug 15;9(8):e105082. doi: 10.1371/journal.pone.0105082 (PMC4134255; doi:10.1371/journal.pone.0105082)
Supplement: Figure S3 — Daily mortality rate of Ternstroemia brasiliensis seedlings per time interval along all the experiment. (DOC) [file pone.0105082.s003.doc]

**
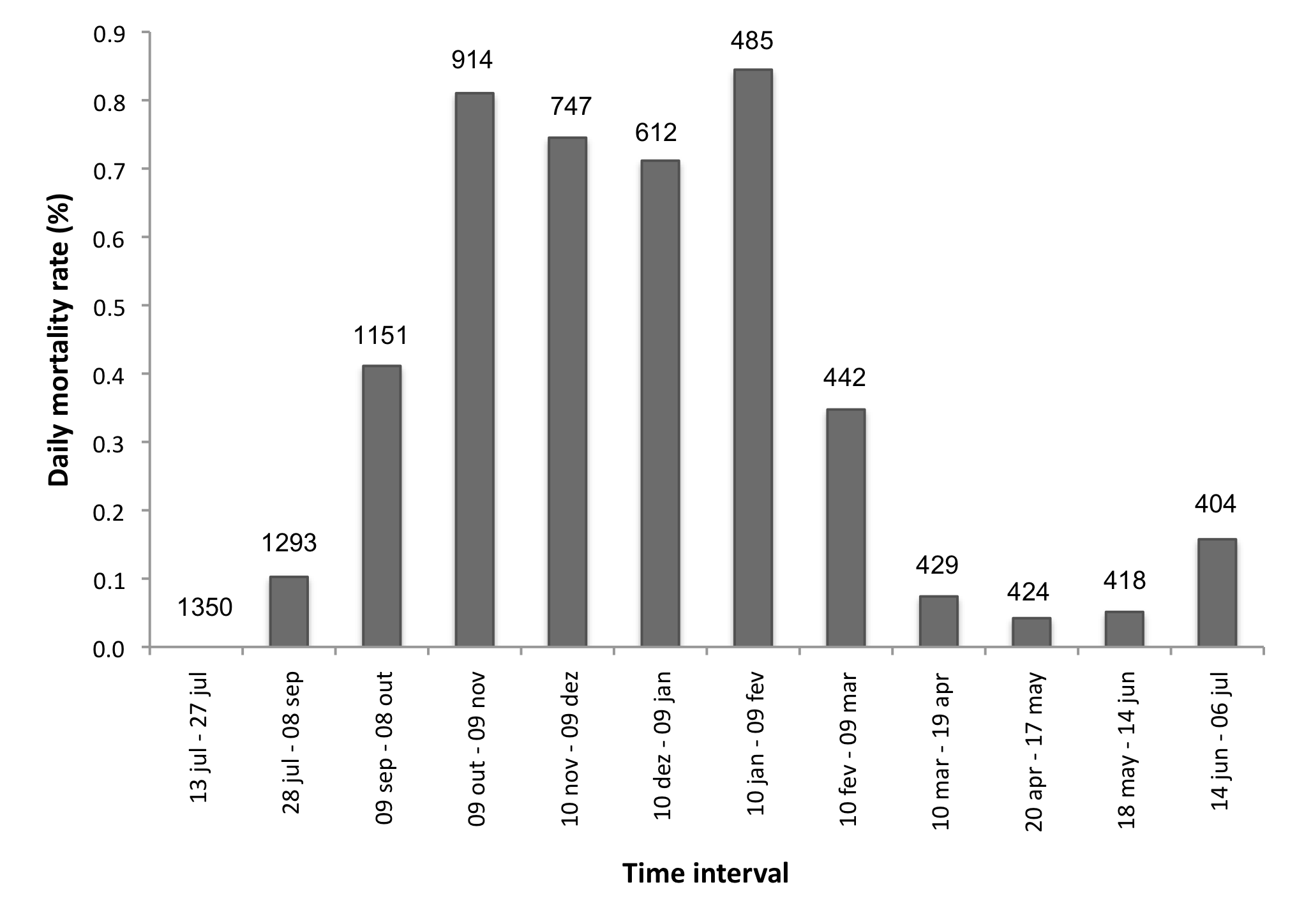
**

**Figure S3** **Daily mortality rate of *Ternstroemia brasiliensis* seedlings per time interval along all the experiment.** Daily mortality rate was calculated as the number of dead seedlings in the interval per the number of seedlings alive in the previous interval per day. The unit of time was considered because the time intervals do not have the same length. Above each bar is the total number of seedlings in the end of each interval.
