# Supplementary material for: Benefit of Shading by Nurse Plant Does Not Change along a Stress Gradient in a Coastal Dune
Source: PLoS One. 2014 Aug 15;9(8):e105082. doi: 10.1371/journal.pone.0105082 (PMC4134255; doi:10.1371/journal.pone.0105082)
Supplement: Figure S4 — Proportion of surviving seedlings of Ternstroemia brasiliensis at three levels of the factor proximity to the seashore (I - closest; III – farthest) and three levels of the factor neighbor. (DOC) [file pone.0105082.s004.doc]

**
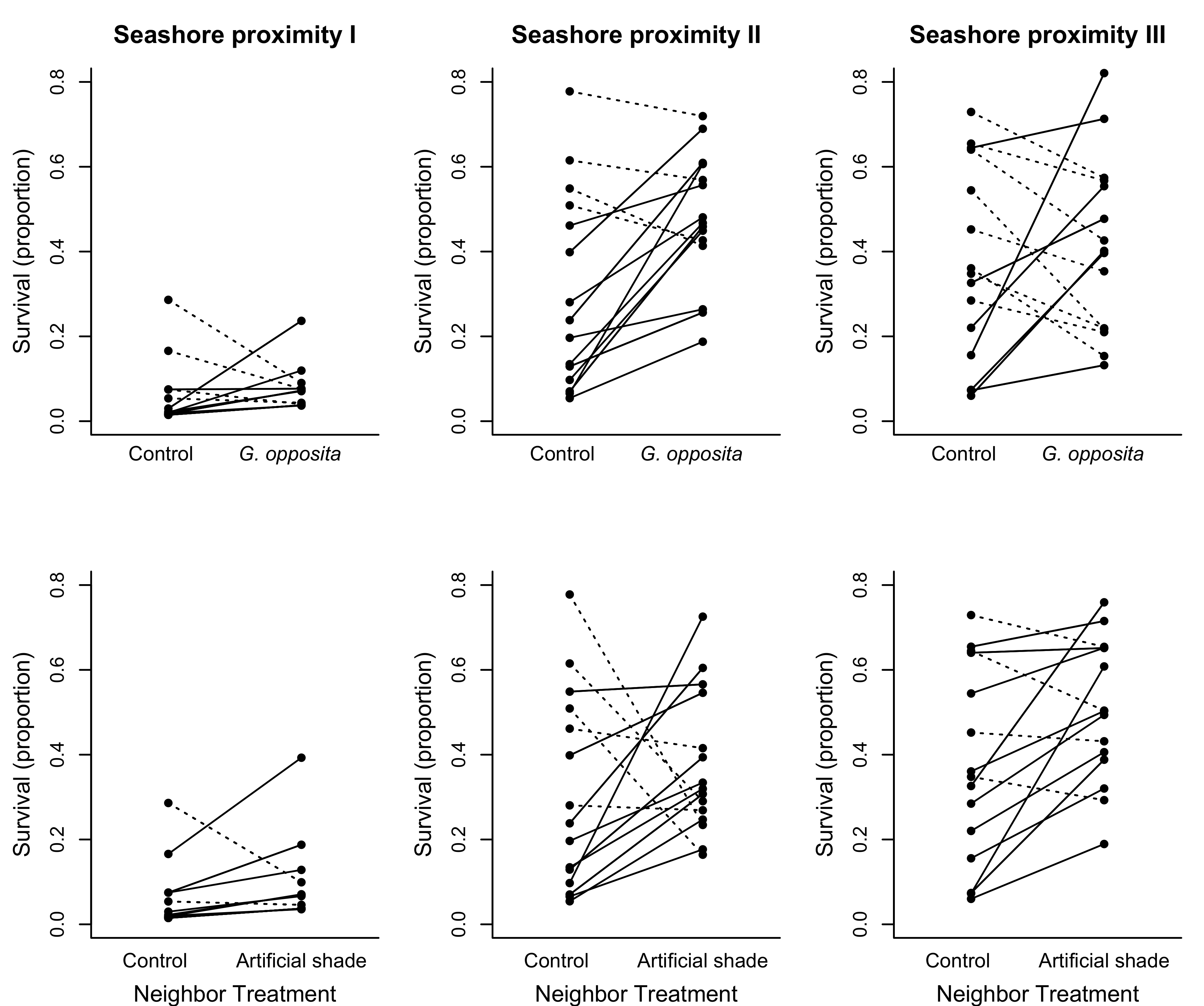
**

**Figure S4 Proportion of surviving seedlings of *Ternstroemia brasiliensis* at three levels of the factor proximity to the seashore (I - closest; III – farthest) and three levels of the factor neighbor (n=15).** The estimates are based on the selected model, which includes the neighbor and proximity to the seashore as fixed variables and the block as a random variable (see model M2.S in Table 1). Circles linked by lines represent levels from the same block. Dashed lines represent blocks where seedlings had better performance in the control, and solid lines represent blocks where seedlings had better performance with a neighbor (*Guapira opposita* or artificial shade).
