# Supplementary material for: Benefit of Shading by Nurse Plant Does Not Change along a Stress Gradient in a Coastal Dune
Source: PLoS One. 2014 Aug 15;9(8):e105082. doi: 10.1371/journal.pone.0105082 (PMC4134255; doi:10.1371/journal.pone.0105082)
Supplement: Figure S5 — Random effects and their 95% interval on survival of Ternstroemia brasiliensis seedlings in the three levels of the neighbor treatment (control, under Guapira opposita and under artificial shade) in each experimental block. (DOC) [file pone.0105082.s005.doc]

**
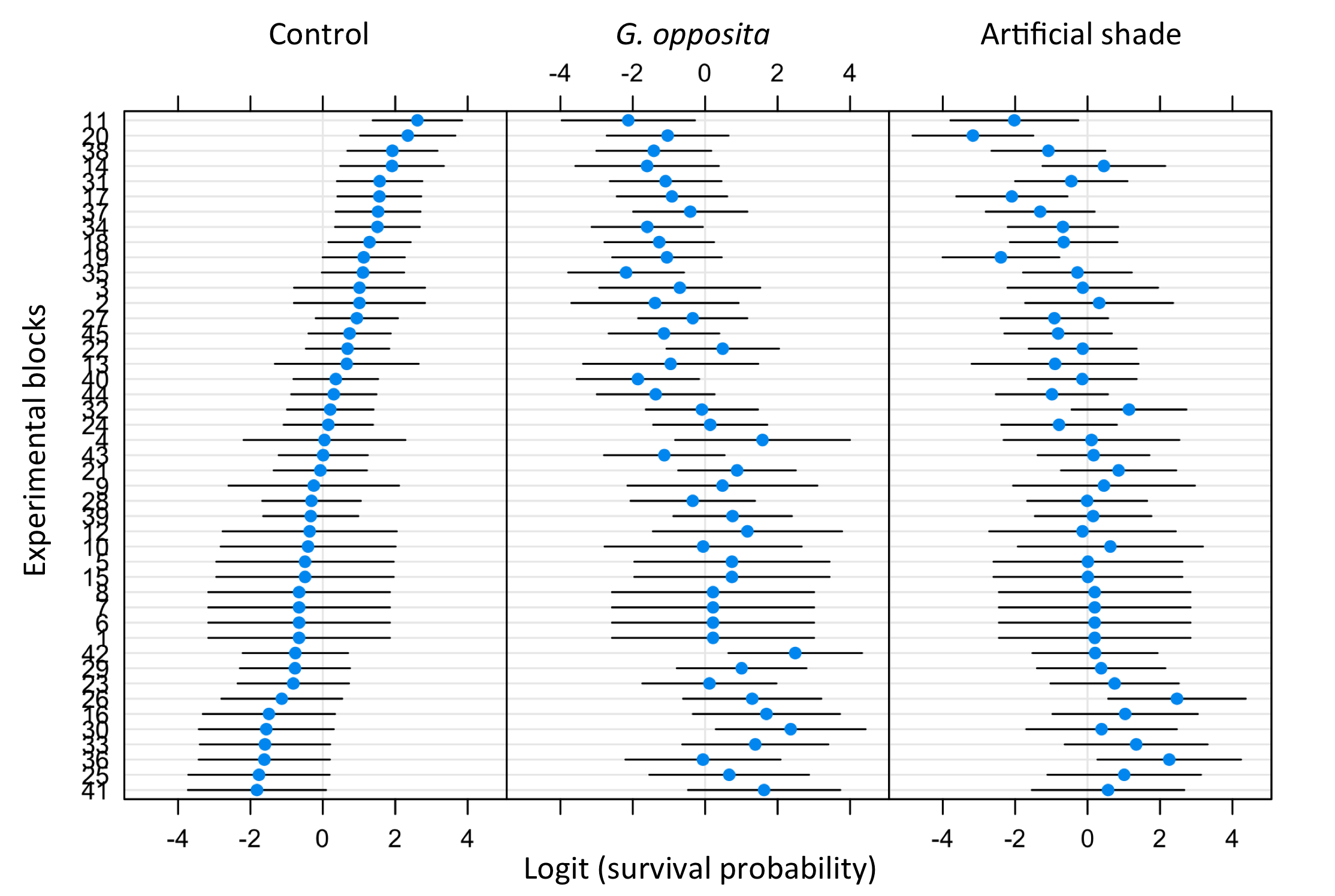
**

**Figure S5** **Random effects and their 95% interval on survival of *Ternstroemia brasiliensis* seedlings in the three levels of the neighbor treatment (control, under *Guapira opposita* and under artificial shade) in each experimental block.** The estimates are based on the selected model, which includes neighbor presence (NE) and distance from the seashore (DI) as the fixed variables and block, including both intercept and slope component, as the random variable (model M2 in Table 1 from the main text). Sub-blocks are ordered from bottom to top according to increasing random effect for the control level. The resulting pattern of the random effects for *G. opposita* and artificial shade reinforces our conclusion that the selected model, which allows for a correlation of the random effects for the control, *G. opposita,* and artificial shade, is suitable. A negative correlation of random effects between the control and the other two neighbor levels indicated that blocks with worse survivorship in the open microsites, on average, benefited more from the presence of a neighbor (*G. opposita* or artificial shade). In contrast, a positive correlation between *G. opposita* and artificial shade levels was found, indicating that blocks with a higher survival of seedlings under *G. opposita* also had good performance under artificial shade.
