# Supplementary material for: Benefit of Shading by Nurse Plant Does Not Change along a Stress Gradient in a Coastal Dune
Source: PLoS One. 2014 Aug 15;9(8):e105082. doi: 10.1371/journal.pone.0105082 (PMC4134255; doi:10.1371/journal.pone.0105082)
Supplement: Table S1 — Results of random component model selection for survival and growth of Ternstroemia brasiliensis seedlings. (DOC) [file pone.0105082.s006.doc]

**Table S1 Results of random component model selection for survival and growth of *Ternstroemia brasiliensis* seedlings.**

|  | Predicted terms included | |  |  |  |
| --- | --- | --- | --- | --- | --- |
| Models | Fixed | Random | K | AIC | Δ AIC |
| Seedlings survival | | | | | |
| **M1.S** | **NE+DI+NE:DI** | **1+NE | Block** | **15** | **301.5** | **0.0** |
| M2.S | NE+DI+NE:DI | 1|Block | 10 | 344.0 | 67.3 |
| Seedlings growth | | | | | |
| M1.G | NE+DI+NE:DI | 1+NE | Block/NE | 22 | 874.4 | 16.1 |
| **M2.G** | **NE+DI+NE:DI** | **1|Block/NE** | **12** | **858.3** | **0.0** |

For survival, the models are general linear mixed models (GLMM) with binomial errors and proportion of surviving seedlings as the response. For growth, the models are linear mixed models (LMM) and increase in aboveground biomass after a year as the response. Presence of neighbor (NE), distance from the seashore (DI), and their interaction (NE: DI) are the fixed variables, and block is the random variable. For growth, the random component still includes the neighbor treatment (NE) nested in each block (Block/NE). The random intercept and slope model (1+NE | Block) was compared with the random intercept model (1| Block). The selected model (Δ AIC< 2) is in bold.
