# Supplementary material for: Benefit of Shading by Nurse Plant Does Not Change along a Stress Gradient in a Coastal Dune
Source: PLoS One. 2014 Aug 15;9(8):e105082. doi: 10.1371/journal.pone.0105082 (PMC4134255; doi:10.1371/journal.pone.0105082)
Supplement: Table S2 — Results of the model selection for soil temperature and photosynthetically active radiation (PAR). (DOC) [file pone.0105082.s007.doc]

**Table S2 Results of random component model selection for soil temperature.**

|  | Predicted terms included | |  |  |  |
| --- | --- | --- | --- | --- | --- |
| Models | Fixed | Random | K | AIC | Δ AIC |
| **M1.T** | **NE** | **1+NE | Block** | **10** | **277.0** | **0.1** |
| **M2.T** | **NE** | **1|Block** | **5** | **276.9** | **0.0** |

The models are linear mixed models (LMM) with presence of a neighbor (NE) as the fixed variable and block as the random variable. The random intercept and slope model (1+NE | Block) was compared with the random intercept model (1| Block). The selected models (Δ AIC< 2) are in bold.
