# Supplementary material for: Benefit of Shading by Nurse Plant Does Not Change along a Stress Gradient in a Coastal Dune
Source: PLoS One. 2014 Aug 15;9(8):e105082. doi: 10.1371/journal.pone.0105082 (PMC4134255; doi:10.1371/journal.pone.0105082)
Supplement: Appendix S1 — Statistical models - full specification, diagnostics and validation. (DOC) [file pone.0105082.s009.doc]

**Appendix S1** **Statistical models - full specification, diagnostics and validation**

Here we present the analysis of the assumptions and full specification of the statistical models used in the study to describe our data: GLMM (generalized linear mixed models) for seedling survival and LMM (linear mixed models) for seedling growth, soil temperature and photosynthetic active radiation (PAR). The analyses of model assumptions follow the recommendations from Pinheiro & Bates (2000), Bolker et al. (2009) and Zuur et al. (2009).

**1. Seedling survival**

**1.1. Specification of the selected model** (model M2.S in Table 1 from the main text)

Y*_ijk_*~ BIN (N=10, p*_ij_*)

logit (p*_ij_*)= (α + a*_i_*)+ β_1_ x SeaProximity II+ β_2_ x SeaProximity III + (β_3_ + b_1_*_ij_*)x G.opposita + (β_3_ + b_3_*_ij_*) x ArtificialShade

a*_i_* ~ N (0, σ_1_^2^) b_1_*_ij_* ~ N (0, σ_2_^2^) b_2_*_ij_* ~ N (0, σ_3_^2^)

*k* = 1,…10 (observation - seedling per treatment combination)

*i* = 1,…45 (experimental blocks)

j = 1,…3 (neighbor levels)

SeaProximity II, SeaProximity III, G.opposita and ArtificialShade are dummy variables that take the value 0 or 1 to indicate the absence or presence of the mentioned categorical effect. Hence, intercept α is the logit of parameter p for seedlings in level I of proximity to the seashore and the control (no shade) level of neighbor treatment.

**1.2. Model validation**

As specified above, the selected model to describe seedling survival includes the effect of the neighbor and the sea proximity as fixed variables; the effect of the block on the intercept and the slope as random variables. As suggested by Bolker et al. (2009), we used Pearson residuals to check overdispersion, which indicates that the model meets the residual deviance criterion (chisp = 44.8; ratio = 0.36; p = 1.00). There is no clear violation of the assumption of normality (Fig. 1A). The residuals are smaller under intermediate fitted values, indicating a lack of variance homogeneity (Fig. 1B). The analysis of the residuals versus each explanatory variable showed minor evidence of heteroscesdasticity as a function of the effect of the neighbor (Fig. 1C) but greater evidence of heteroscesdasticity as a function of seashore proximity, with smaller variance in the proximity class I than proximity classes II and III (Fig. 1D). However, the residuals versus fitted values by neighbor and seashore proximity classes showed that the pattern observed in Fig. 1A was observed in most of the treatment combinations (Fig. 2), indicating that the pattern observed in Fig. 1B is not completely explained by heteroscesdasticity between groups defined by fixed effects. In any case, the correlation between the residuals and the fitted values observed in Fig. 1B and the heteroscesdasticity of the seashore proximities groups (Fig. 1D) could affect the validation of the model. To check if these data properties affected the validation of our conclusions, we used weighted modeling to overcome the heteroscesdasticity, a procedure often used in linear models (Pinheiro and Bates 2000; Zuur et al. 2009). Adopting this procedure, we evaluated a model weighted by the precision, giving more weight to the observations with smaller variances. The weights were estimated from a loess model (local polynomial regression fitting) fitted to the relationship between the absolute residuals and fitted values (red curve in Fig. 1B). From the weighted full model (including both fixed factors and their interaction), we reran the same model selection procedure. We found that, as in the previous model selection, the more plausible model was the one that included neighbor and proximity to the sea as fixed effects and the block as a random component. This result showed that the variance heterogeneity did not affect our conclusions regarding the importance of the neighbor and the proximity to the sea on seedling survival. The weighted model generated estimates for the effect of *G. opposita* and artificial shading that were a little higher than those from our model; hence, our model is conservative regarding the effect of the fixed variables. Finally, the assumption of normality for the random effects seems reasonable (Fig. 3), though there is some evidence of long tails at both ends of the distribution for the artificial shade slope (Fig. 3C). In conclusion, model M2.S does not violate any assumption in a way that changes our conclusions about the importance of the neighbor and the proximity to the seashore on seedling survival.

**Fig. 1** Model validation plots for model M2.S (model specification is in topic 1.1). A) Normal quantile-quantile plot of standardized residuals (solid red line: expected if residuals are normally distributed; dashed red lines: 95% bootstrap confidence intervals); B) Fitted values versus absolute residuals (solid red line: loess fit); C) Standardized residuals versus the level of neighbor fixed effect; D) Standardized residuals versus the level of seashore proximity fixed effect

**
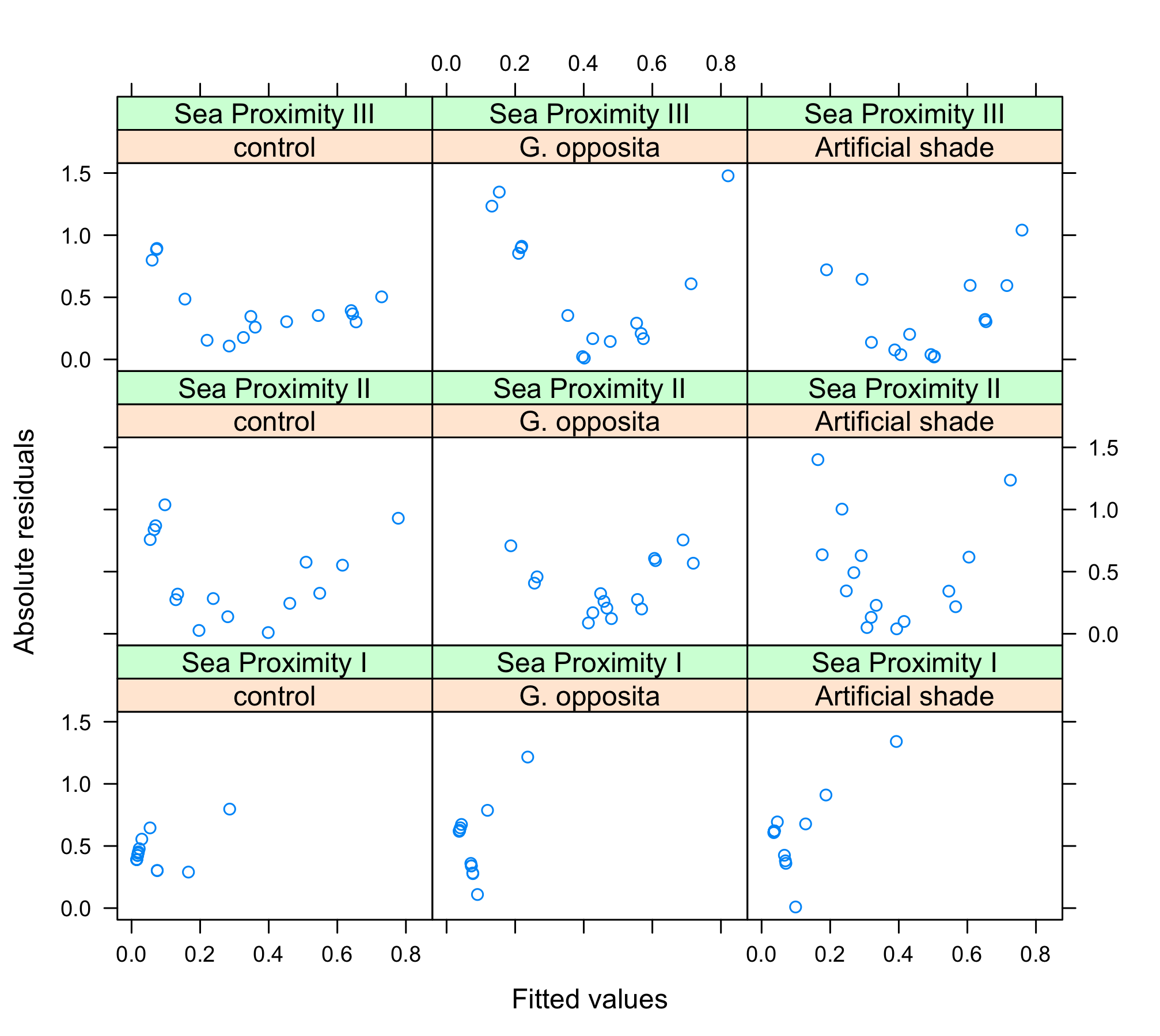
**

**Fig. 2** Fitted values versus absolute residuals for model M2.S by the levels of neighbor and seashore proximity (both fixed effects)

**
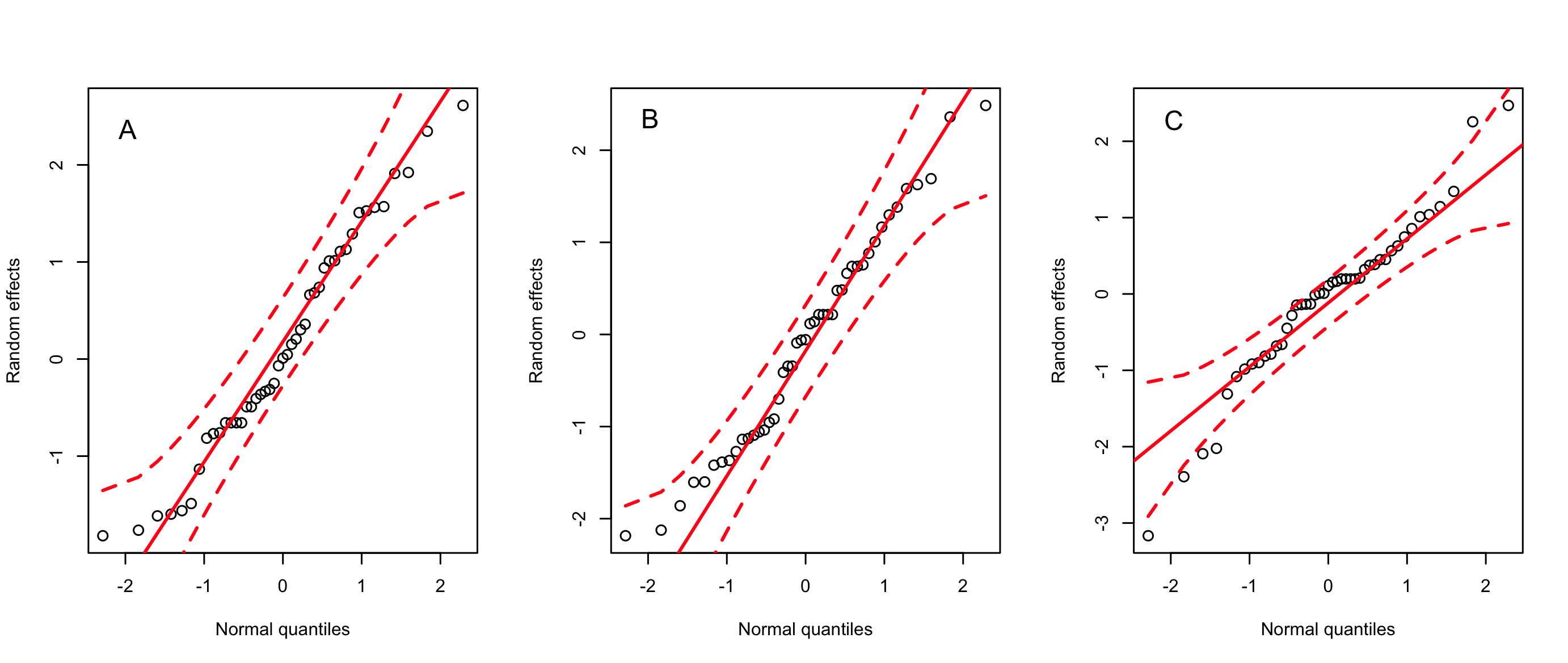
**

**Fig. 3** Normal quantile-quantile plots of estimated random effects for model M2.S. Solid red lines represent the expected values if the random effects are normally distributed; dashed red lines represent the 95% bootstrap confidence intervals. A) Random effects on the intercept (a*_i_* in the previous model specification); B) Random effects for the effect of *G. opposita* (b_1_*_ij_* ); C) Random effect for the effect of artificial shade (b_2_*_ij_*)

**2. Seedling growth**

**2.1. Specification of the selected model** (model M3.G in Table 1 from the main text)

*y_ijkl_*= β*_j_* + b*_i_* + b*_ij_* + ε*_ijkl_*

b*_i_* ~ N (0, σ_1_^2^) b*_ij_* ~ N (0, σ_2_^2^) ε*_ijkl_* ~ N (0, σ_l_^2^)

*k* = 1,…10 (observation - seedling per treatment combination)

*i* = 1,…41 (experimental blocks)

j = 1,…3 (neighbor levels)

*l* = 1,…3 (seashore proximity)

**2.2. Model validation**

The first full model of the seedling growth included neighbor, seashore proximity and their interaction as fixed variables and random effects at two levels: the effects of the block and the effect of the neighbor nested in each block (seedling growth ~ neighbor + seaproximity + neighbor * seaproximity + (1|Block/neighbor). By examining the assumptions of this model, we realized that the variability in seedling growth was greater in the sea proximity level III than in the other two classes (Fig. 4), violating the assumption of homogeneity of the within-error group.

**Fig. 4** Standardized residuals versus seashore proximity fixed effect for the following model with no weighted variances: seedling growth ~ neighbor + sea proximity + neighbor * seaproximity + (1|Block/neighbor)

To account for the heteroscesdasticity, we weighted our model using a variance structure function that allows different variances for each level of seashore proximity. The comparison between the two models indicated that the model with weighted variances was much better than the model without weighted variance (ΔIAC= 712 for the non-weighted model). Therefore, we reran the model selection to infer the importance of the fixed effects using weighted variances (see the results in Table 1 in the main text). As described in topic 2.1, the selected model included the effect of the neighbor as a fixed effect and the block and the neighbor within the block as the random effects (model M3.G in Table 1 in the main text). The following are the analyses of the assumptions of this selected model. Regarding the assumption of normality, there is evidence of long tails at both ends of the distribution (Fig. 4A). Regarding the variances, there is a range of fitted values where no observation was conducted, but for the other parts of the range, the spread of the residuals is roughly similar (Fig. 4B). The analysis of the residuals versus each explanatory variable showed no evidence of heteroscesdasticity for the levels of neighbor treatment (Fig. 4C) and minor evidence for the levels of seashore proximity, with higher variance in proximity level III (Fig. 4D). Finally, the assumption of normality for both random effects seems reasonable (Fig. 5 and 6), though there is some evidence of a long tail at the negative end of the distribution for the random effect of the block due a single observation (Fig. 5). In conclusion, model M3.G seems to roughly meet the linear mixed model assumptions and consequently is suitable for analyzing seedling growth data.


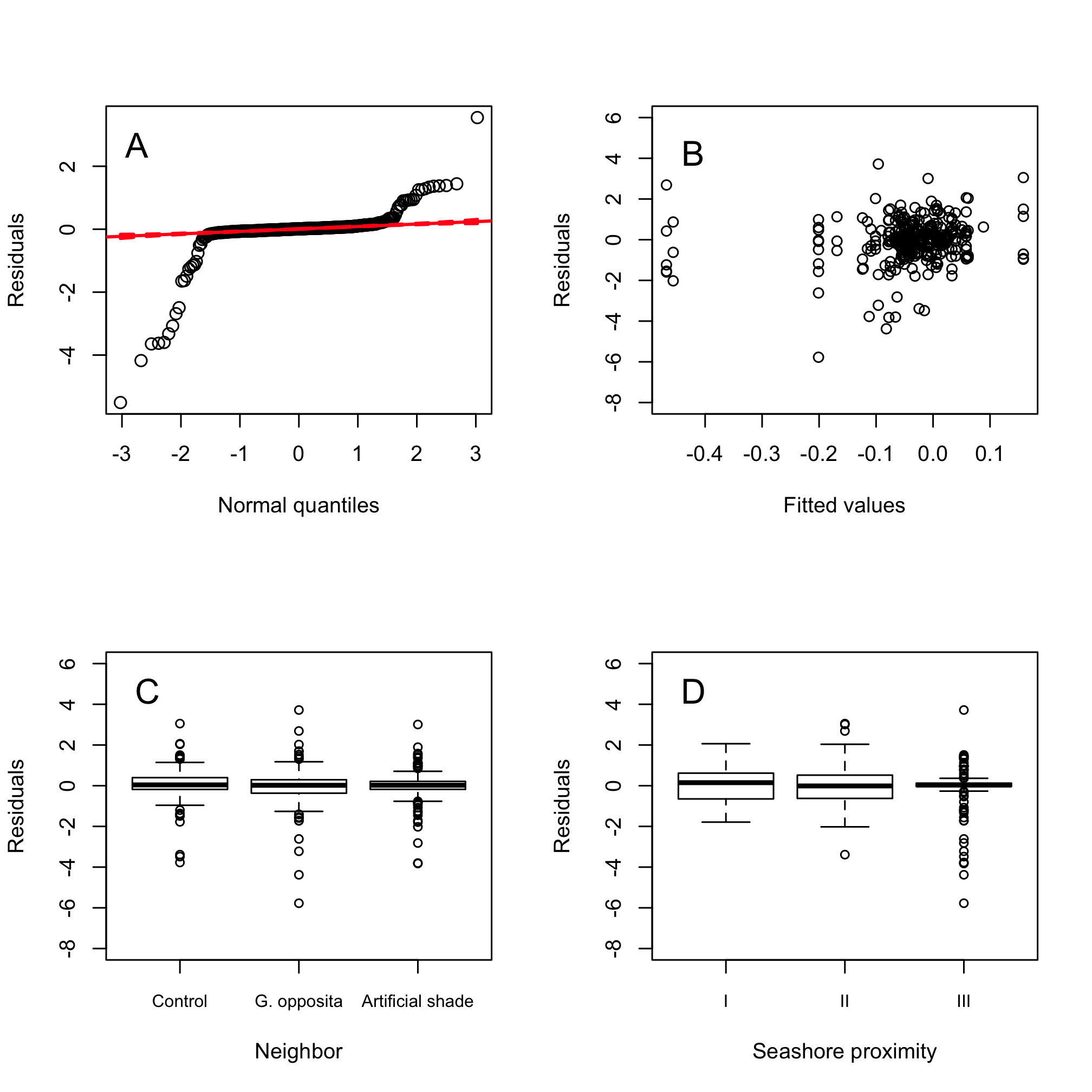


**Fig. 4** Model validation plots for model M3.G in Table 1 of the main text (model specification is in the topic 2.1). A) Normal quantile-quantile plot of standardized residuals (solid red line: expected if residuals are normally distributed; dashed red lines: 95% bootstrap confidence intervals); B) Fitted values versus standardized residuals; C) Standardized residuals versus levels of neighbor fixed effect; D) Standardized residuals versus levels of seashore proximity fixed effect


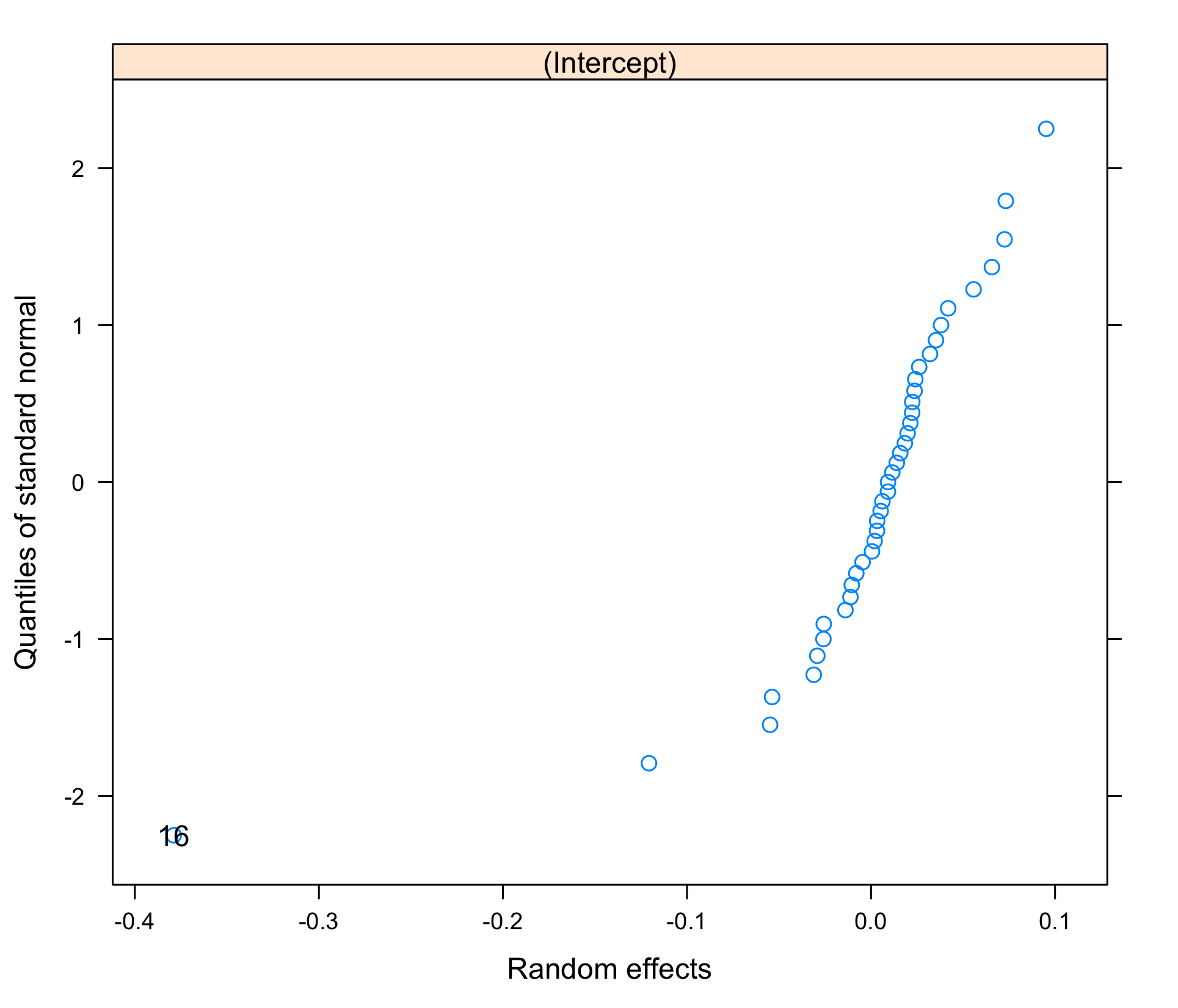


**Fig. 5** Normal quantile-quantile plot of the estimated random effects of the blocks for the M3.G model

**Fig. 6** Normal quantile-quantile plot of the estimated random effects of the neighbor within each block for the M3.G model

**3. Soil temperature**

**3.1. Representation of the selected model** (model M1.T in Table 3 from the main text)

*y_ij_*= β*_j_* + b*_i_* + ε*_ij_*

b*_i_* ~ N (0, σ_1_^2^) ε*_ij_* ~ N (0, σ_2_^2^)

*i* = 1,…18 (experimental blocks)

j = 1,…3 (neighbor levels)

**3.2. Model validation**

As detailed above, the selected model (model M1.T in Table 3 from the main text) includes the effect of the neighbor as a fixed variable and the effect of the block on the intercept as a random variable. There is minor evidence of residual non-normality with a heavier tail in the left part of the distribution (Fig. 7A). The spread of the residuals seems roughly the same across the range of fitted values (Fig. 7B), indicating homogeneity of the variances. However, the analysis of the residuals versus the explanatory variable neighbor showed minor evidence of heteroscesdasticity, with higher variance for the *G. opposita* neighbor treatment than for artificial shade or the control (Fig. 7C). To test the heteroscesdasticity of this model, we compared it with another model with different variances for each level of the neighbor factor. The comparison by AIC indicated that the model with no weighted variance is as plausible as the model with weighted variance (ΔAIC = 1.0), suggesting that the distinct variances observed in Fig. 7C do not violate the assumption of variance homogeneity. There is no evidence that the distribution of the estimated random effects is not normal (Fig. 7D). In conclusion, model MT.1 seems to meet the linear mixed model assumptions and consequently is suitable for analyzing soil temperature data.

**
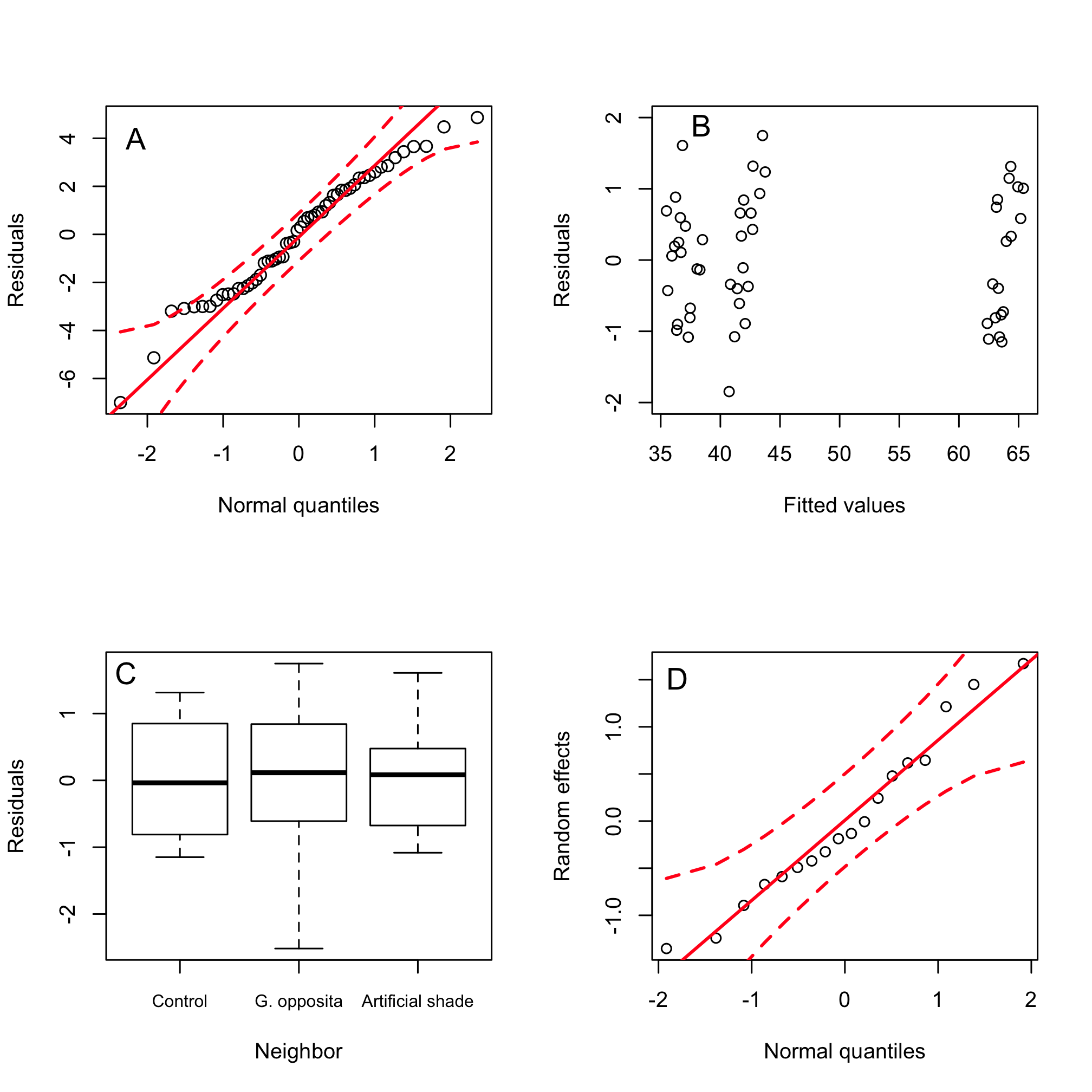
**

**Fig. 7** Validation plots for model M1.T (model specification is in topic 3.1). A) Normal quantile-quantile plot of standardized residuals (solid red line: expected if residuals are normally distributed; dashed red lines: 95% bootstrap confidence intervals); B) Fitted values versus standardized residuals; C) Standardized residuals versus the level of neighbor fixed effect; D) Normal quantile-quantile plot of the estimated random effects

**4. Photosynthetically active radiation (PAR)**

**4.1. Specification of the selected model** (model M1.P in Table 3 from the main text)

*y_ij_*= β*_j_* + b*_i_* + ε*_ij_*

b*_i_* ~ N (0, σ_1_^2^) ε*_ij_* ~ N (0, σ_2_^2^)

*i* = 1,…18 (experimental blocks)

j = 1,…3 (neighbor levels)

**4.2. Model validation**

As specified in the previous topic, the selected model (model M1.P in Table 3 from the main text) includes the effect of the neighbor as a fixed variable and the effect of the block on the intercept as the random variable. There is no strong evidence of residual non-normality, but there are long tails at both ends of the distribution (Fig. 8A). The spread of the residuals is not the same across the range of fitted values, indicating variance heterogeneity (Fig. 8B). In fact, the analysis of the residuals versus neighbor showed smaller variance for the control level of the neighbor treatment than for *G. opposita* and artificial shade (Fig. 8C). To test the heteroscesdasticity of this model, we tried to compare it with another model with different variances for each level of the neighbor factor. However, the model with weighted variance had problems with numerical convergence. Although the violation of homogeneity assumption could invalidate the conclusion of the statistical analysis, we strongly believe that it does not because the PAR values are actually very different between the control level and the other two neighbor levels. In fact, the difference is so high (see Table 4 in the main text) that a statistical analysis could even be unnecessary. Finally, regarding the random effects, there is no evidence that the distribution of the estimated random effects is not normal (Fig. 8D). In conclusion, we believe that the homogeneity violation detected in this model validation does not affect our biological conclusion that the photosynthetic active radiation (PAR) is higher in the exposed areas (control) than beneath the shade of *G. opposita* or artificial structures.

**Fig. 8** Model validation plots for model M1.P (model specification is in topic 4.1). A) Normal quantile-quantile plot of standardized residuals (solid red line: expected if residuals are normally distributed; dashed red lines: 95% bootstrap confidence intervals); B) Fitted values versus standardized residuals; C) Standardized residuals versus the level of neighbor treatment; D) Normal quantile-quantile plot of the estimated random effects

**5. ReferenceS**

Bolker BM, Brooks ME, Clark CJ, et al. (2009) Generalized linear mixed models: a practical guide for ecology and evolution. Trends Ecol Evol 24:127–135.

Pinheiro JC, Bates DM (2000) Mixed-Effects models in S and S-Plus. Springer-Verlag, New York.

Zuur AF, Ieno EN, Walker NJ, Saveliev AA, Smith, GM (2009) Mixed effects models and extensions in ecology with R. Springer, New York
